# A method to determine pharmacokinetic parameters based on andante constant-rate intravenous infusion

**DOI:** 10.1038/s41598-017-13437-6

**Published:** 2017-10-16

**Authors:** Rui-hong Yu, Yong-xiao Cao

**Affiliations:** Department of Pharmacology, Health Science Center, Xi’an Jiaotong University, Xi’an, China

## Abstract

On account of the disturbance from the distribution phase, the concentration-time curve of drugs cannot fully reflect the characteristics of elimination, and thus, it is difficult for present methods to obtain ideal pharmacokinetic parameters. This paper presents a method to determine pharmacokinetic parameters based on an andante constant-rate intravenous infusion. A mathematical model of the constant-rate intravenous infusion combined with the elimination of first-order kinetics was established. During infusion, the accumulation tendency of drugs was deduced as ${C}_{t}={C}_{{0}}+({C}_{ss}-{C}_{{0}})\cdot (1-{e}^{-Kt})$ using the principle of calculus. Then, the method to determine the pharmacokinetic parameters was summed up. After collecting the blood drug concentration (*C*_*t*_) -time (*t*) data from a constant-rate (*v*) infusion period, an exponential regression analysis was conducted to obtain the elimination rate constant (*K*) and plateau concentration (*C*_*ss*_). Then, the half-life (*t*_*1/2*_), apparent volume of distribution (*V*_*d*_) and clearance rate (*CL*) were calculated based on the equations, *t*_*1/2*_ = 0.693/*K*, *V*_*d*_ = (*v*/*K*)/*C*_*ss*_ and *CL* = *v*/*C*_*ss*_, respectively. In addition, an application example of cimetidine in a beagle dog was used to demonstrate the implementation process of the method.

## Introduction

Pharmacokinetics is the process that determines the drug amount in the body over time, which is essential for understanding the pharmacodynamics of a drug. It is a study of drug disposition in the body and focuses on the changes in the blood drug concentration. The pharmacokinetic characteristics can be quantitatively expressed by its parameters, such as the elimination rate constant (denoted as *K*), half-life (*t*_*1/2*_), apparent volume of distribution (*V*_*d*_) and total clearance rate (*CL*). Pharmacokinetic study is mainly performed by the compartmental or non-compartmental analysis. The non-compartmental analysis is the most popular method in pharmacokinetic studies at the present time. We statistically analyzed two hundred literatures about the pharmacokinetics of certain drugs, which were published in 2015 in PubMed scope and found that non-compartmental analysis accounted for greater than 95 percent of the total studies.

The noncompartmental analysis is similar to kinetic analyses used in other scientific disciplines, such as chemical kinetics and chromatographic theory, both of which are analyzed basing on statistical moments principles.^1^ The noncompartmental method evaluates the exposure of a drug by estimating the area under the curve (*AUC*) and the moment curve (*AUMC*) of a drug concentration-time graph, which is more versatile in that it relies very little on the compartmental model or the *in vivo* process of the drugs. The compartmental method estimates the concentration-time graph using kinetic models. A number of functional models have been developed in order to simplify the study of pharmacokinetics, and these models are based on the consideration of the organism as a number of related compartments.

In fact, the models do not always truly reflect the real situation within the body in the compartmental model analytics. Incredibly, the number of compartments for the same drug will not be unique when the administration route or the sample schedule is different.^2^ The noncompartmental method estimates the elimination rate constant and half-life by performing a linear regression of the logarithmic drug concentration-time data in the terminal phase.^3,4^ However, the terminal phase is only a part of the elimination phase. Some deviations can appear when the slope of the terminal phase is used to extrapolate the slope of the whole elimination phase. In addition, the range of the terminal elimination phase involved in the regression is specified by the individual user, which leads to an arbitrary estimation, and becomes even worse if the error is high.^5,6^ In the other hand, the mean residence time (*MRT*) determined by the *AUMC/AUC* merely demonstrates the characteristic of the central compartment or blood, but it cannot sufficiently present the drug exposure in the whole organism. As a result, the equation *t*_*1/2*_ = 69.3%·*MRT* is false other than for drugs of the one-compartment model. Even though mainstream pharmacokinetic analysis has transformed gradually from the compartmental model to the noncompartmental model in recent years, the obtained pharmacokinetic parameters are still less accurate. Its ultimate cause is the interference of the distribution phase, especially for drugs of the bi- or multi-compartment model, and thus, it is difficult to figure the pure elimination phase out to determine the pharmacokinetic parameters.

Here, we propose a novel method to determine pharmacokinetic parameters, which is based on an andante constant-rate intravenous infusion, and the disturbance from the distribution phase is expected to be avoided.

## Results

### The relationship between the drug amount in the body and time during the intravenous infusion

In constant-rate intravenous infusion, drugs are delivered into the body at a constant speed, *v*. Assuming that elimination follows first-order kinetics, the elimination rate of a drug is proportional to the total drug amount in the body. When infusion starts, the rate of drug administration is much greater than the elimination rate. Therefore, the total amount of drug in the body increases rapidly, while the elimination amount is little. Over time, drug accumulation in the body still continues, and the rate and amount of elimination increases gradually. As a result, the accumulation rate of a drug decreases gradually. Eventually, when the rate of elimination equals the infusion rate, the drug amount in the body no longer accumulates and reaches a steady state, i.e., a plateau.

To analyze the accumulation trend of the total drug amount over time, we utilized a fundamental theorem of calculus to integrate the two processes of infusion and elimination. The constant-rate intravenous infusion is regarded as infinite times (*n*) of intravenous bolus injections with an equal dose and zero interval. Then, the infusion duration (0 ∼ *t*) can be divided into *n* subintervals of an equal width (*dt* = *t*/n), 1^st^ *dt*, 2^nd^ *dt*, 3^rd^ *dt*, …, *n* ^th^ *dt*.

It is supposed that the drug is eliminated in accordance with first-order kinetics with an elimination rate constant, *K*. When a drug is intravenously injected into the body, at time *t*, the drug amount, *A*, in the body can be calculated as:

$$A={A}_{{0}}\cdot {{\rm{e}}}^{-Kt}$$

where *A*_0_ is the initial drug amount in the body and is equal to the injection dosage.

**Figure 1:**
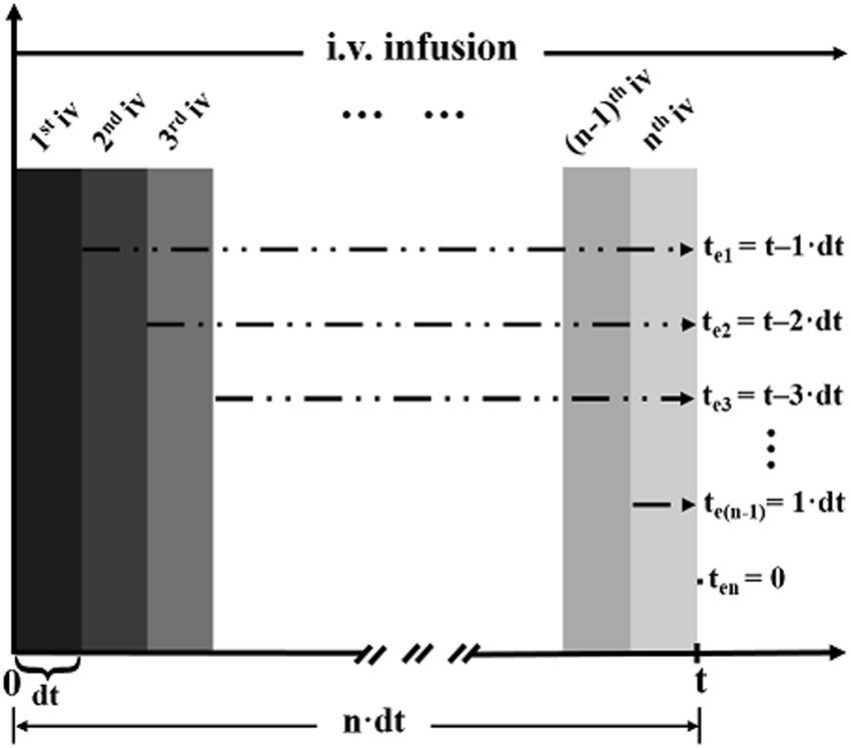
Schematic model of an integral analysis of the constant-rate intravenous infusion. The constant-rate intravenous infusion is divided into *n* times of intravenous bolus injections. The elimination time of every bolus is individually defined as ${t}_{ei}=t-i\cdot dt$. For example, the elimination time of the 1^st^ bolus, *t*_*e1*_, is equal to $(t-1\cdot dt)$ and that of the last or *n* ^th^ bolus, *t*_*en*_ is zero on account of $t=n\cdot dt$.

The initial amount of each bolus equals the administration dosage and can be expressed as:

$${A}_{{0}}=v\cdot dt$$

where *dt* is infinitely close to zero numerically. Therefore, the beginning of elimination for every bolus is intended to be the time point of every bolus as exactly accomplished. As shown in Fig. 1, at a certain time, *t*, during the infusion, the elimination time of the 1^st^ bolus (*t*_*e1*_) is expressed as:

$${t}_{e{1}}=t-dt$$

The elimination time of the 2^nd^ bolus (*t*_*e2*_) is as follows:

$${t}_{e{2}}=t-2dt$$

And that of the 3^rd^ bolus (*t*_*e3*_) will be:

$${t}_{e{3}}=t-3dt$$

The elimination time of the (*n*−1)^th^ bolus (*t*_*e(n−1)*_) will be:

$${t}_{e(n-1)}=t-(n-1)dt=1\cdot dt$$

The elimination time of the last or *n* ^th^ bolus (*t*_*en*_) is zero, i.e.,

$${t}_{en}=t-ndt=0$$

Therefore, at a certain time, *t*, during the infusion, the residual amount of the drug from 1^st^ bolus (*A*_*r1*_) can be expressed as:

$${A}_{r{1}}=(v\cdot dt)\cdot {{\rm{e}}}^{-K(t-dt)}$$

Similarly, the residual amount of the drug from the 2^nd^ bolus (*A*_*r2*_) is expressed as:

$${A}_{r2}=(v\cdot dt)\cdot {{\rm{e}}}^{-K(t-2dt)}$$

The residual amount of the drug from the 3^rd^ bolus (*A*_*r3*_) is:

$${A}_{r{3}}=(v\cdot dt)\cdot {{\rm{e}}}^{-K(t-3dt)}$$

The residual amount of the drug from the (*n*−1)^th^ bolus (*A*_*r(n-1)*_) will be:

$${A}_{r(n-1)}=(v\cdot dt)\cdot {{\rm{e}}}^{-K[t-(n-1)dt]}$$

And the residual amount of the drug from the *n* ^th^ bolus (*A*_*rn*_) is expressed as:

$${A}_{rn}=(v\cdot dt)\cdot {{\rm{e}}}^{-K(t-ndt)}$$

Lumping them together, the total drug amount (*A*_*t*_) in the body at time *t* can be expressed as:

$$\begin{array}{rcl}{A}_{t} & = & {A}_{r1}+{A}_{r2}+{A}_{r3}+\cdots +{A}_{r(n-1)}+{A}_{rn}\\  & = & v\cdot dt\cdot {{\rm{e}}}^{-K(t-1dt)}+v\cdot dt\cdot {{\rm{e}}}^{-K(t-2dt)}+v\cdot dt\cdot {{\rm{e}}}^{-K(t-3dt)}+\cdots \\  &  & +v\cdot dt\cdot {{\rm{e}}}^{-K[t-(n-1)dt]}+v\cdot dt\cdot {{\rm{e}}}^{-K(t-ndt)}\\  & = & v\cdot dt\cdot {{\rm{e}}}^{-Kt}\cdot ({{\rm{e}}}^{K\cdot 1dt}+{{\rm{e}}}^{K\cdot 2dt}+{{\rm{e}}}^{K\cdot 3dt}+\cdots +{{\rm{e}}}^{K\cdot (n-1)dt}+{{\rm{e}}}^{K\cdot ndt})\\  & = & v\cdot {{\rm{e}}}^{-Kt}\cdot \sum _{({\rm{i}}=1)}^{{\rm{n}}}{{\rm{e}}}^{(i\cdot K\cdot dt)}\cdot dt\end{array}$$

According to the definition of a definite integral^7^, the definite integral of *A*_*t*_ from 0 to *t* is:

$$\begin{array}{ccc}{A}_{t} & = & v\cdot {{\rm{e}}}^{-Kt}\cdot \mathop{{\rm{l}}{\rm{i}}{\rm{m}}}\limits_{{\rm{n}}\to {\rm{\infty }}}{\sum }_{(i=1)}^{n}{{\rm{e}}}^{(i\cdot K\cdot dt)}\cdot dt\\  & = & v\cdot {{\rm{e}}}^{-Kt}\cdot {\int }_{(t)}^{0}{{\rm{e}}}^{Kt}\cdot dt\end{array}$$

After a change of variable^8^, the equation above can be solved based on the theorem, $\int {{\rm{e}}}^{x}\cdot dx={{\rm{e}}}^{x}+c$ ^9^ and yields:

1 $${A}_{t}=v\cdot {{\rm{e}}}^{-Kt}\cdot [1/K\cdot ({{\rm{e}}}^{Kt}+c)],{\rm{in}}\,{\rm{which}}\,c\,{\rm{is}}\,{\rm{a}}\,{\rm{constant}}\,{\rm{value}}.$$

However, at the beginning of a continuous infusion, the drug amount in the body is little. Only when the drug amount in the body reaches a certain level, i.e., the threshold value, can the elimination organs begin to eliminate the drug. Here, we denote the threshold drug amount in the body as *A*_*0*_. Because there is hardly elimination during this period, the accumulation speed of the drug amount is rapid, and the accumulation duration for the drug amount up to *A*_*0*_ is extremely short, which can be ignored. Thus, we define the moment when the drug amount in the body reaches *A*_*0*_ as 0 on the timeline.

As a part of the total drug amount in the body, *A*_*0*_ is also involved in elimination after the above pre-defined time of 0. At time *t*, the residual amount of the *A*_*0*_ (*A*_*r0*_) is as follows:

$${A}_{r{0}}={A}_{{0}}\cdot {{\rm{e}}}^{-Kt}$$

Then, the relational expression of the drug amount in the body over time, i.e., equation 1, should be revised to:

2 $${A}_{t}=v\cdot {{\rm{e}}}^{-Kt}\cdot [1/K\cdot ({{\rm{e}}}^{Kt}+c)]+{A}_{{0}}\cdot {{\rm{e}}}^{-Kt}$$

Based on the reality mentioned above, when *t* = 0, the drug amount (*A*_*t*_) at that moment is equal to *A*_*0*_, so that

$$\begin{array}{rcl}{A}_{{0}} & = & v\cdot {{\rm{e}}}^{-K\cdot {0}}\cdot [1/K\cdot ({{\rm{e}}}^{K\cdot {0}}+c)]+{A}_{{0}}\cdot {{\rm{e}}}^{-K\cdot {0}}\\  & = & v/K\cdot (1+c)+{A}_{{0}}\end{array}$$

Only when the value of constant *c* is equal to −1 can the above function hold. Therefore, equation 2 can be expressed as:

$${A}_{t}=v\cdot {{\rm{e}}}^{-Kt}\cdot [1/K\cdot ({{\rm{e}}}^{Kt}-1)]+{A}_{{0}}\cdot {{\rm{e}}}^{-Kt}$$

This equation can be rearranged into:

3 $${A}_{t}={A}_{{0}}+(v/K-{A}_{{0}})\cdot (1-{{\rm{e}}}^{-Kt})$$

In equation 3, *t* refers to the elapsed infusion time from the beginning. *A*_*t*_ denotes the total drug amount in the body at a corresponding time *t*. *v* is the constant rate of the intravenous infusion, and *K* is the elimination rate constant.

When *t* tends towards infinity, a steady-state/plateau amount (*A*_*ss*_) of a drug can be determined as:

4 $${A}_{ss}={A}_{{0}}+(v/K-{A}_{{0}})\cdot (1-0)=v/K$$

When *v*/*K* is substituted by *A*_*ss*_, equation 3 can be changed as:

5 $${A}_{t}={A}_{{0}}+({A}_{ss}-{A}_{{0}})\cdot (1-{{\rm{e}}}^{-Kt})$$

**Figure 2:**
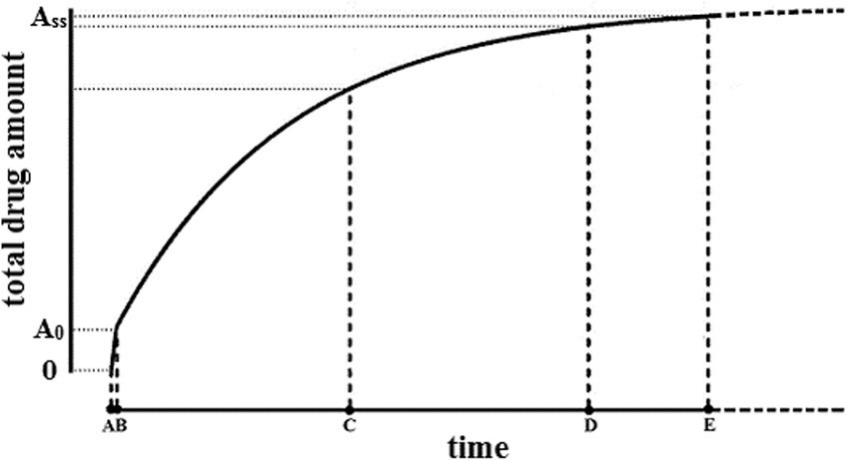
Drug accumulation curve during an andante constant-rate intravenous infusion. (**A**) in the absence of elimination, the initial rise in the drug amount is extremely rapid during a remarkably short period. (**B**) when the drug amount reaches *A*_*0*_, elimination starts. (**C**) and (**D**) the drug amount in the body accumulates exponentially. E, the steady-state equilibrium is nearly reached. Note that *t*_*A*_ and *t*_*B*_ are particularly close to each other on the timeline.

In addition, the process of drug accumulation expressed by equation 5 can be simply schematically modeled, as shown in Fig. 2.

### The relationship between blood drug concentration and time during intravenous infusion

Due to the complexity of drug distribution, the total drug amount in the body is impossible to get measured. Commonly, in distribution equilibrium, the change in the drug amount in different organs or tissues occurs at the same rate. Therefore, the amount of drug in the blood is assumed to be proportional to the amount in the body, so is the blood drug concentration. Generally, the proportionality between the blood drug concentration and the total drug amount in the body is defined as the apparent volume of distribution (*V*_*d*_). The andante intravenous infusion is a continuous administration mode with a microdose per unit of time, while the distribution of the drugs from the blood to periphery runs rapidly. Thus, the distribution is completed immediately for every microdose, and from the overall view, the drug distribution is basically balanced during i.v. infusion.

Therefore, equation 5 can be demonstrated as:

$${C}_{t}\cdot {V}_{d}={C}_{{0}}\cdot {V}_{d}+({C}_{ss}\cdot {V}_{d}-{C}_{{0}}\cdot {V}_{d})\cdot (1-{{\rm{e}}}^{-Kt})$$

After reduction of a fraction, the relationship between the drug concentration in the blood and the corresponding time is:

6 $${C}_{t}={C}_{{0}}+({C}_{ss}-{C}_{{0}})\cdot (1-{{\rm{e}}}^{-Kt})$$

In equation 6, *C*_*ss*_ refers to the drug concentration of the steady state in blood. *C*_0_ refers to the threshold concentration for elimination. *K* is the elimination rate constant. *t* refers to the elapsed infusion time from the beginning, and *C*_*t*_ denotes the drug concentration in the blood at the corresponding time *t*.

Equation 6 can be rearranged logarithmically into a straight line equation.

7 $$\mathrm{ln}\,({C}_{ss}-{C}_{t})=\,\mathrm{ln}\,({C}_{ss}-{C}_{{0}})-Kt$$

For a certain drug, under intravenous infusion, the logarithm of the difference between the plateau concentration and the real-time concentration is linearly associated with time.

### The calculation of the pharmacokinetic parameters

Equation 6, the relational expression of the blood drug concentration and time during an andante constant-rate intravenous infusion, was fitted to a one phase association model in exponential regression, which is expressed as a uniform expression as follows:

$$y={y}_{{0}}+({\rm{plateau}}-{y}_{0})\cdot (1-{{\rm{e}}}^{-Kx})$$

Specific to equation 6, *t* is an independent variable, and *C*_*t*_ is a dependent variable. *C*_*0*_, *C*_*ss*_ and *K* are constant and can be obtained from an analytic result after the exponential regression of the concentration-time data from the intravenous infusion period.

From the above, the intravenous infusion rate *v* is foregone, and the elimination rate constant *K*, the steady-state concentration *C*_*ss*_, and the threshold concentration for elimination *C*_*0*_ can be obtained through a concentration-time data analysis. Next, other relevant pharmacokinetic parameters, including *t*_*1/2*_, *V*_*d*_, and *CL*, are then calculated using the following equations:

8 $${t}_{{1}/{2}}=0.693/K$$

9 $${V}_{d}={A}_{ss}/{C}_{ss}=(v/K)/{C}_{ss}$$

10 $$CL=K\cdot {V}_{d}=v/{C}_{ss}$$

Note that the calculation of *V*_*d*_ in equation 9 is based on the steady state. In this extreme moment, both the drug amount in the body (equation 4) and the drug concentration in the blood can be predicted.

### The determination of pharmacokinetic parameters of cimetidine

In the example experiment conducted in a beagle dog, the plasma cimetidine concentration of samples during a constant-rate infusion were assayed by HPLC and are presented in Table 1.

**Table 1:** Plasma cimetidine concentration-time data during the andante constant-rate intravenous infusion in a beagle dog.

time (h)	cimetidine (μg·ml^−1^)
0.17	1.38
0.5	1.88
1.0	2.31
1.5	2.83
2.0	2.96
3.0	3.73
4.0	4.41
5.0	4.27
6.0	4.42
7.0	4.41
7.5	4.34
8.0	4.06

**Figure 3:**
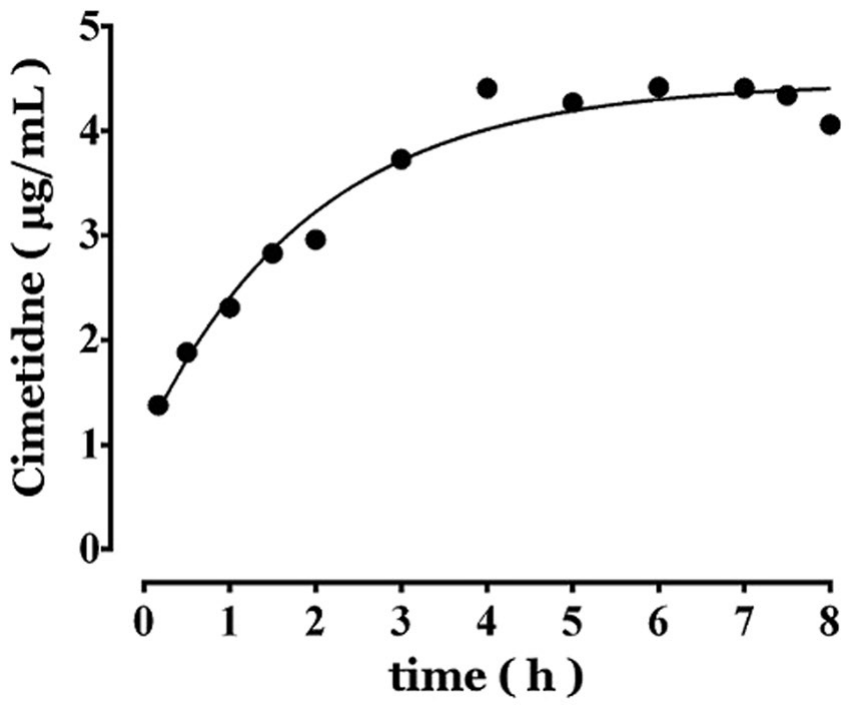
Plasma cimetidine concentration-time curve during a constant-rate intravenous infusion in a beagle dog.

Based on equation 6, ${C}_{t}={C}_{{0}}+({C}_{ss}-{C}_{{0}})\cdot (1-{{\rm{e}}}^{-Kt})$, the plasma concentration-time data of the cimetidine during intravenous infusion in Table 1 was exponentially regressed. The regressed plasma concentration-time curve of cimetidine is presented in Fig. 3. The correlation coefficient was 0.9709, indicating that there exists a close exponential correlation between the plasma concentration of cimetidine and the time during the andante constant-rate intravenous infusion.

The function between the plasma cimetidine concentration and time during intravenous infusion is expressed as:

$${C}_{t}=1.04+3.42\cdot (1-{{\rm{e}}}^{-0.51\cdot t})$$

and from the analytic result or the above function, *C*_*0*_, *C*_*ss*_ and *K* were obtained as follows:

$${C}_{{0}}=1.04\,{\rm{\mu }}{\rm{g}}\cdot {{\rm{mL}}}^{-1}$$

$${C}_{ss}=4.46\,{\rm{\mu }}{\rm{g}}\cdot {{\rm{mL}}}^{-1}$$

$$K=0.51\,{{\rm{h}}}^{-1}$$

In addition, the infusion rate, *v* was calculated based on the amount and duration of the infusion

$$v=282.2\,{\rm{mg}}/8\,{\rm{h}}=35.28\times {10}^{3}\,{\rm{\mu }}{\rm{g}}\cdot {{\rm{h}}}^{-1}$$

Based on equations 8–10, thus:

$${t}_{{1}/{2}}=0.693/K=0.693/0.51\,{{\rm{h}}}^{-1}=1.36\,{\rm{h}}$$

$${V}_{d}=(v/K)/{C}_{ss}=(35.28\times {10}^{3}\,{\rm{\mu }}{\rm{g}}\cdot {{\rm{h}}}^{-1}/0.51\,{{\rm{h}}}^{-1})/4.46\,{\rm{\mu }}{\rm{g}}\cdot {\rm{mL}}=15.51\,{\rm{L}}$$

$$CL=v/{C}_{ss}=35.28\times {10}^{3}\,{\rm{\mu }}{\rm{g}}\cdot {{\rm{h}}}^{-1}/4.46\,{\rm{\mu }}{\rm{g}}\cdot {{\rm{mL}}}^{-1}=7.91\,{\rm{L}}\cdot {{\rm{h}}}^{-1}$$

Finally, the *t*_*1/2*_, *V*_*d*_, and *CL* of cimetidine in beagle dog were determined as 1.36 h, 15.51 L, and $7.91\,{\rm{L}}\cdot {{\rm{h}}}^{-1}$, respectively.

## Discussion

In the intravenous infusion period, the blood drug concentration rises exponentially, and all the blood concentration-time data can be applied to define the accumulation curve of a drug on account of the free disturbance from the distribution phase. Here, *K* and *t*_*1/2*_ were calculated based on the whole elimination period, which is hidden in the exponential accumulation process of the drug during the intravenous infusion. However, in the non-compartmental analysis, the determination of *K* and *t*_*1/2*_ are based on the terminal elimination phase. Due to the existence of experimental errors, the slope of the terminal phase in a natural logarithm concentration-time profile should not be extrapolated to the entire elimination phase. In the new method, *V*_*d*_ was calculated basing on the total drug amount in the body and the blood drug concentration of the steady state in which the distribution of the drug is balanced, i.e., *V*_*d*_ = *A*_*ss*_/*C*_*ss*_. Then, *CL* was calculated as $CL=K\cdot {V}_{d}$ in the new method. However, in the non-compartmental analysis, *CL* is estimated through the AUC, which is an interim parameter that is related to the entire concentration-time curve, in which the unbalanced distribution phase is included. Additionally, in the non-compartmental analysis, the value of *V*_*d*_ cannot be determined directly because it is difficult to obtain the total drug amount in the body at any moment. As a result, two approximate concepts, *V*_*z*_ and *V*_*ss*_, are derived from *V*_*d*_ in the non-compartmental analysis, and indirect methods for the calculation of *V*_*z*_ and *V*_*ss*_ are introduced. *V*_*z*_ denotes the distribution volume calculated through the terminal phase, i.e., *V*_*z*_ = *CL*/*K*. V_ss_ denotes the distribution volume calculated through the so-called steady state, i.e., ${V}_{ss}=CL\cdot MRT$, in which *MRT* is calculated as *MRT* = *AUMC*/*AUC* and is inevitably disturbed by the unbalanced distribution phase, just as the AUC. Because of the uncertainty of *K*, *CL* and *MRT*, the *V*_*z*_ and *V*_*ss*_ determined in the non-compartmental analysis are probably incorrect. By contrast, the pharmacokinetic parameters determined by the new method are more credible.

The potential limitations of the new method include three points. First, the new method is applicable solely for drugs with an injection preparation, but it could be expanded into a pharmacokinetic study system, which would be applicable for all kinds of dosage forms. The core of the pharmacokinetic study system is to achieve distribution equilibrium through a sustained mode of administration. Second, the method requires a relative longer time of administration, which may cause some inconvenience. Third, a zero-order/constant rate drug input is required.

Cimetidine was used as an application example to show the implementation process of the new method. Obviously, the analysis of the drug concentration-time data in the new method is only involved in an exponential regression, which can be conducted with a universal software such as *GraphPad Prism*, *Origin* or *SPSS*. In addition, the calculations of the pharmacokinetic parameters are simple and only require a few manual calculations, and the rationale of calculation formulas is easy to understand.

To completely describe the blood drug accumulation trend of infusion, the intravenous infusion of cimetidine in the application example continued up to 8 h, which is over five half-lives of cimetidine. In fact, *C*_*ss*_ is a constant in equation 6 and can be predicted based on the multiple drug concentration-time data through exponential regression. Furthermore, *A*_*ss*_ is calculated as *A*_*ss*_ = *v*/*K*. Therefore, in the actual pharmacokinetic research using the new method, it is not necessary to infuse over five half-lives to achieve steady state.

The new method presented here can be used to determine the pharmacokinetic parameters for experimental animals and clinical patients. In addition, this method allows for the optimization of an individualized dosage regimen. For example, the estimation of the infusion rate required to produce the desired maximum blood concentration, especially for drugs with a narrow safety range.

In conclusion, the study found an andante constant-rate intravenous infusion method to determine the pharmacokinetic parameters without disturbance from the distribution phase. It can be used for experimental and clinical pharmacokinetic research.

## Methods

### Establishment of a mathematical model

In the andante constant-rate intravenous infusion, the accumulation amount of a drug in the body equals the difference in the infusion and elimination amounts. The delivery speed of the constant-rate intravenous infusion is a foregone value, based on which the drug amount administered into body can be calculated. Most drugs are eliminated in accordance with first-order kinetics. Therefore, the mathematical model of the drug amount in the body during a constant-rate infusion is a combination of two regular processes, a constant-rate import (the constant rate is denoted as *v*) and a first-order kinetic export (the rate constant is denoted as *K*). When a drug is intravenously infused into the body in andante, its distribution in the body is basically balanceable, i.e., the drug concentration in blood is proportional to the total drug amount in the body. Therefore, the relationship of the blood drug concentration and time during the andante constant-rate infusion is the same as that of the drug amount in the body and time.

### Application example

Cimetidine was used as an application example to show the implementation process of the new method. This study was carried out in strict accordance with the recommendations in the Guide for the Care and Use of Laboratory Animals of the National Institutes of Health. The experimental protocols for using a beagle dog were approved by the Animal Ethics Committee at Xi’an Jiaotong University, Xi’an, China. One beagle dog weighing 12 kg crouched on a customized holder and received a 282.2 mg constant-rate infusion of cimetidine for 8 h through the right brachiocephalic vein. Blood samples were collected during infusion from the left brachiocephalic vein.

The samples were centrifuged at 4 °C at 3000 r/min for 10 min. The plasma was then transferred to a new tube and stored upright at −20 °C until sample preparation for analysis. The plasma sample (200 μL), internal standard (ranitidine, 100 μg/mL, 10 μL) and 15% perchloric acid (100 μL) were added sequentially and mixed by vortexing for 1 min. After centrifugation, the resulting supernatant was injected into the chromatographic system (Dionex Corporation, Sunnyvale, USA) for analysis. The concentrations of cimetidine in the samples were determined using a validated high-performance liquid chromatography (HPLC) assay method, in which samples were analyzed under reverse-phase conditions with a mobile phase of acetonitrile/pH = 3 sodium phosphate buffer (8/92, vol/vol) and detected through ultraviolet detector with a wavelength of 225 nm. The lower limit of quantitation was 0.1 μg/mL for cimetidine, and a calibration curve was linear over the range of 0.1–10 μg/mL. The interday assay accuracy, expressed as relative error, ranged from −2.1% to 6.4% in the QC samples with three levels, and the assay precision, expressed as relative standard deviation, ranged from 3.3% to 4.6%. In addition, the recovery and stability of cimetidine were also within the accepted criterion.

### Data analysis

The relational expression of the drug amount versus time during the constant-rate intravenous infusion was deduced using the fundamental theorem of calculus.

The data analysis in the application example was performed by *GraphPad Prism 6.0*. The analysis was operated by successively choosing nonlinear regression—exponential subtype—one-phase association mode.
